# Tropical Medicine

**Published:** 1905-05-13

**Authors:** 


### Tropical Medicine.

It is one of the disadvantages of the system under which hospitals are conducted in this country that it is apt to give rise to an apparent rivalry between institutions which are directed to the attainment of a common end, but which are compelled to seek for support from the public by dwelling upon the results of their own work in such a manner as apparently to do less than justice to the work of others. We have lately seen, for example, something approaching to a dispute concerning the relative merits of the respective Schools of Tropical Medicine of London and of Liverpool, institutions animated by the same spirit, working upon similar lines, striving for the attainment of the same ends, and both conferring incalculable benefits not only upon seamen, colonists, and colonial traders, but also upon the nation as a whole by removing dangers from the prosecution of commercial enterprise in extensive portions of the world. Both alike owe their origin primarily to considerations which were urged upon Mr. Chamberlain, when he was Secretary of State for the Colonies, by Sir Patrick Manson, the medical adviser to the Colonial Office, and, secondarily, to the rare sagacity which enabled a political official to turn an attentive ear and an open mind to representations which were purely scientific in their character, and to perceive the extent and the magnitude of their bearings upon the national welfare.

The general result of the operations of the two schools, and of the labours of the men sent out by them, has been not only to increase enormously the power of medicine to deal in a curative fashion with many serious and dangerous diseases, but also to afford good grounds for hoping that many of these diseases will fall within the reach of preventive measures, and that countries in which it has until recently been impossible for Europeans to live and work in safety may before long be rendered as salubrious as our own. In taking this general view of the question, we desire to dissociate ourselves entirely from any sort of comparison between the London and the Liverpool institutions, or from any attempt to reckon up their respective contributions towards the attainment of ends which are common to both. It is, therefore, with no forgetfulness of the merits of Liverpool that we direct attention to the appeal which the Duke of Marlborough, as chairman of the London School, has put forward on its behalf, with the hope not only of paying off the debt of about £6,000 by which it is at present hampered, but also with that of raising sufficient money to enable its work to keep pace with the constant increase of the demands which are made upon its resources. The Duke reminds us that the import and export trade of the Crown colonies and India amounts approximately to £400,000,000 sterling per annum, and that, as an increasing number of our kith and kin are annually required to carry on the work in the tropics, it is undoubtedly a matter of imperial importance that an increasing knowledge of the diseases from which they are liable to suffer or to perish should be made an earnest object of our endeavours. Among the results of this kind already obtained by the staff or the pupils of the school are some to which the term epoch-making may without exaggeration be applied.

The students of the school are taught not by lecture only, but by actual demonstration of tropical diseases, with their causes and the best methods of treatment. The students actually examine the germs, follow their life histories, and become enabled to recognise them in practice and to apply the proper treatment. The commoner and many of the rarer tropical diseases are watched by the students in the wards of the hospital which adjoins the school. It is on behalf of both that the Duke asks for support, and mentions that a banquet in aid of the conjoined institutions will be held at the Hotel Cecil before these words fall under the eyes of our readers. We would remind any who may not be present that it will be none the less easy to forward contributions to the offices of the school, at 13a Cockspur Street, S.W.

